# A summarization approach for Affymetrix GeneChip data using a reference training set from a large, biologically diverse database

**DOI:** 10.1186/1471-2105-7-464

**Published:** 2006-10-23

**Authors:** Simon Katz, Rafael A Irizarry, Xue Lin, Mark Tripputi, Mark W Porter

**Affiliations:** 1 Gene Logic Inc., 610 Professional Dr, Gaithersburg, MD, 20876, USA; 2 Department of Biostatistics, Johns Hopkins Bloomberg School of Health, 615 N Wolfe St, Baltimore, MD, 21205, USA; 3 Department of Applied Mathematics and Statistics, Johns Hopkins. University, 302 Whitehead Hall, 3400 North Charles Street, Baltimore, MD, 21218, USA

## Abstract

**Background:**

Many of the most popular pre-processing methods for Affymetrix expression arrays, such as RMA, gcRMA, and PLIER, simultaneously analyze data across a set of predetermined arrays to improve precision of the final measures of expression. One problem associated with these algorithms is that expression measurements for a particular sample are highly dependent on the set of samples used for normalization and results obtained by normalization with a different set may not be comparable. A related problem is that an organization producing and/or storing large amounts of data in a sequential fashion will need to either re-run the pre-processing algorithm every time an array is added or store them in batches that are pre-processed together. Furthermore, pre-processing of large numbers of arrays requires loading all the feature-level data into memory which is a difficult task even with modern computers. We utilize a scheme that produces all the information necessary for pre-processing using a very large training set that can be used for summarization of samples outside of the training set. All subsequent pre-processing tasks can be done on an individual array basis. We demonstrate the utility of this approach by defining a new version of the Robust Multi-chip Averaging (RMA) algorithm which we refer to as refRMA.

**Results:**

We assess performance based on multiple sets of samples processed over HG U133A Affymetrix GeneChip^® ^arrays. We show that the refRMA workflow, when used in conjunction with a large, biologically diverse training set, results in the same general characteristics as that of RMA in its classic form when comparing overall data structure, sample-to-sample correlation, and variation. Further, we demonstrate that the refRMA workflow and reference set can be robustly applied to naïve organ types and to benchmark data where its performance indicates respectable results.

**Conclusion:**

Our results indicate that a biologically diverse reference database can be used to train a model for estimating probe set intensities of exclusive test sets, while retaining the overall characteristics of the base algorithm. Although the results we present are specific for RMA, similar versions of other multi-array normalization and summarization schemes can be developed.

## Background

Pre-processing of Affymetrix GeneChip^® ^feature-level data has been a widely researched topic over the past few years. Many of the commonly used algorithms utilize models where parameters are estimated using data from multiple arrays. These approaches are typically used in the normalization and summarization steps. Examples of multi-array procedures are RMA, gcRMA, MBEI, and, most recently, PLIER [1-4]. Each of the algorithms have been extensively compared to one another based on a variety of dilution and spike-in series of data sets [5-8]. From these studies, measures of precision and accuracy have been utilized to determine advantages and disadvantages for each of these methods. In general, multi-array based methods outperform those that derive expression measures using data from just the array in question.

A problem associated with these algorithms that has not received much attention is the limitation they impose on data archiving. When data from a new study becomes available, all arrays are pre-processed together to obtain expression measures. Because different data is used to define the normalization routine and estimate probe-effects, data from different studies might not be comparable because of this pre-processing bias. A solution is to group together the feature-level data for both experiments and re-run the pre-processing algorithm. However, this approach will be logistically impossible in the case of building large probe set-level reference databases. These databases can be used to quickly combine samples that may have been generated from a variety of different experiments into a user-defined data set based on some common attribute such as clinical or pathological status, treatment level, or technology-derived attribute. The lack of comparability between sets of samples normalized based on different schemes does not allow for archiving and continual updating of probe set-level data and ultimately prevents the analyst from combining disparately normalized samples into cohesive sets.

There is a need to develop summarization schemes that offer both the statistical advantages of multi-array algorithms such as RMA, gcRMA, MBEI, and PLIER and the flexibility of a workflow that is not specific to a single set of samples. We utilize the RMA model as an example methodology to demonstrate the feasibility of such an approach,

We have utilized an alternate RMA workflow that includes two distinct steps: (a) training of an RMA model based on a large number of biologically distinct Affymetrix GeneChip samples from Gene Logic's BioExpress reference database and (b) application of the resulting RMA model parameters to multiple test sets. We have named this alternate workflow, "Reference RMA" (refRMA), to emphasize the reliance of this methodology on a standardized training set of samples exclusive of a test set. Results in this paper are shown for the HG U133A Affymetrix GeneChip array for a specific implementation of refRMA trained using 1,614 .cel files representing 144 different organ types from four different pathology states. We show that the application of such a biologically diverse model to test data results in similar probe set-level data when compared to classic RMA outputs as measured by overall data structure, correlation, differential expression, and other metrics. Furthermore, a model trained on a large training set of biologically distinct samples seems to be robust for tissues that were not used in its training. Finally, we show that the model can be applied to data sets external to the site of microarray processing specific for the training set such that the model can be used universally.

## Results

### Training set for the "Full refRMA" model

Two goals for building a common reference refRMA model are to incorporate as much biological variability as possible into the training set and to appropriately balance the principal sources of variability such that there is not a large degree of overrepresentation of any one of the types. The Gene Logic BioExpress reference database is comprised of thousands of samples from 144 tissues and cell types, which are represented in up to four pathological categories (i.e., Normal, Diseased, Malignant, or Benign) as determined by a board certified pathologist's review of each sample. Individual brain regions were treated as separate organs due to their highly heterogeneous gene expression profiles.

Selection of the samples was based on a two-step filtering system. The first step involved an initial balancing protocol that attempted to correct for unintended effects on the normalization due to organs that are disproportionately represented within the database. For instance, samples from organs such as liver, lung, and kidney have high numbers of samples, whereas samples from organs of more limited research may only have a few samples. A balanced pool of samples was created by capping the maximum samples per organ and pathological category at 20 samples and randomly selecting samples for those organs with sample numbers above the cap. This first filter resulted in a balanced pool of over 6,000 samples. The next filter randomly selected 1,500 non-blood samples from the balanced pool in order to accommodate computational memory limitations.

Whole blood was considered a special case due to its unique expression profiles as compared with other tissue types, its relative importance in clinical genomics, and the usage of two types of blood processing protocols that have or have not undergone globin depletion procedures. The final number of samples used for the training set was 1,614 after the addition of 114 whole blood samples available at the time of model training. A summarized list of the individual organ or cell types, their pathological status as determined by a board certified pathologist, and the number of samples within each category is provided as a supplemental file; see Additional file 1. A total of 251 tissue and pathological categories are present within the training set over the 144 represented organ and cell types.

### Results of Affycomp as a comparative benchmark

**Figure 1:**
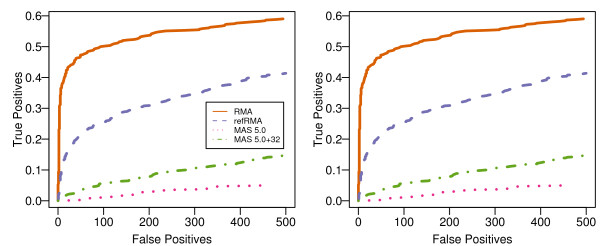
**ROC curves for the Classic RMA, Full refRMA, and MAS5 algorithms via affycomp**. Each of the summarization algorithms are shown with respect to False Positive probe sets vs. True Positive percentage for the affycomp spiked-in HG U133A data set. The spiked-in probe sets used are limited to a) lowly expressed probe sets (≤ 2 pM) and b) moderately expressed probe sets (≥ 4 and ≤ 32 pM) as defined in [9]. The Full refRMA model performs better than either of the MAS5 algorithms, but does not do as well as the Classic RMA model for the spiked-in probe sets. The likely reason for this result is discussed in the text.

The Full refRMA model trained as described above was used to summarize HG U133A arrays with spiked-in probe sets as part of the commonly used affycomp comparative benchmark system [9]. This system uses a variety of measures and diagnostic plots to assess ability of summarization methods to accurately and precisely estimate the nominal fold changes as a function of gene expression levels. Full refRMA was compared with Classic RMA, where the 42 spiked-in samples served as both training and test set, original MAS5, and MAS5+32 algorithms. The MAS5+32 algorithm adds a constant of 32 to the resulting MAS5 values in order to stabilize variance. Results of the comparative assessment are presented in Table 1 and Figure 1.

**Table 1:** Results of affycomp comparative benchmark.

**Metric**	**Optimal Value**	**Full refRMA**	**Classic RMA**	**MAS 5.0**	**MAS 5.0+32**
**Null log-fc IQR**	0.00	0.20	0.13	0.47	0.34
**Null log-fc 99%**	0.00	0.51	0.29	2.83	1.30
**Null log-fc 99.9%**	0.00	0.74	0.40	4.01	1.81

**Low AUC**	1.00	0.20	0.45	0.00	0.03
**Med AUC**	1.00	0.53	0.87	0.00	0.01
**High AUC**	1.00	0.67	0.92	0.00	0.01
**Weighted Avg AUC**	1.00	0.28	0.55	0.00	0.03

**Low slope**	1.00	0.27	0.25	0.61	0.42
**Med slope**	1.00	0.68	0.69	0.70	0.67
**High slope**	1.00	0.87	0.82	0.81	0.81

Specific descriptions of each metric are provided in the original publication describing the affycomp system [9].

Table 1 shows that, for measures that capture areas under the ROC curves (AUC's) and percentiles of fold changes for non-spiked genes, refRMA performs better than either of the MAS5 algorithms, but does not perform as well as Classic RMA for this data set. Figure 1 shows the low and medium probe set expression ROC curves from which the AUC's were derived. The advantage of refRMA over single-chip summarization algorithms is obvious in this plot. In addition, refRMA outperformed the other algorithms for regression between nominal and observed fold changes at higher levels of expression, as evidenced by the high expression intensity slope measure (i.e., "High slope") in Table 1.

The overall interpretation from the affycomp results is that refRMA outperforms MAS5 summarization, but does not perform as well as Classic RMA where the training set and test set are identical. This is to be expected under normal circumstances given that Classic RMA is highly specific to its training set. The spiked-in nature of the experiment introduces an additional probe-level effect into the system and most likely contributes to the size of the disparity between Classic and refRMA, as Classic RMA will be trained on this effect and refRMA will not. These results indicate a limitation to the applicability of refRMA to data that has been heavily manipulated. The manipulation may be due to exogenous addition of probe, such as in the case of a spike-in data set, or may be due to other sample processing factors such as alternate amplification and labelling processes. In addition, the data suggests that a refRMA model trained on a large, biologically diverse set of samples may not summarize highly tissue-specific probe sets as well as Classic RMA, although refRMA may outperform single chip-based summarization methods such as MAS5.

### Application of refRMA to true biological samples

**Figure 2:**
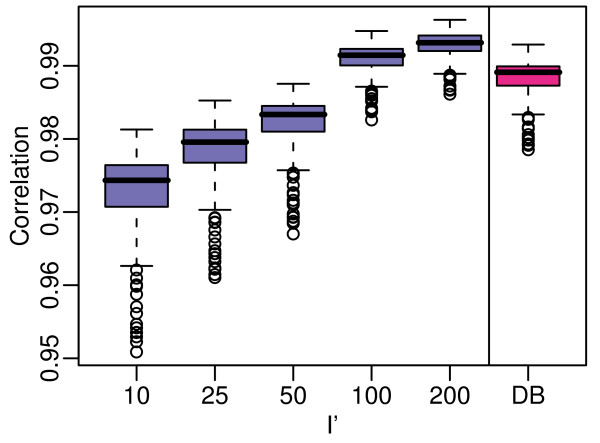
**Correlations between Classic and refRMA data trained with GEO samples**. Randomly selected samples from normal GEO samples of mutiple organs were used to train refRMA models at each of the sample sizes (***I***) indicated and were applied to 50 exclusive test set samples. Correlation for each test sample summarized by refRMA was calculated relative to the same test sample summarized by Classic RMA (i.e., trained on the 50 test set samples) using all probe sets. The mean correlation across the 50 test samples was then calculated. This process was repeated 100 times using random selection of both training and test sets to yield the correlation distributions shown as box plots. The entry shown as "DB" is the Full refRMA model trained on 1,614 samples from Gene Logic's reference database. Note that the GEO-based models, where test set experiments are not completely exclusive of training set, show slightly higher correlations than the Full refRMA model, which represents complete exclusivity of training and test sets.

To test the performance of the Full refRMA model on unmodified biological samples, a variety of normal control samples from multiple organs were extracted from the Gene Expression Omnibus (GEO) database based on the descriptive fields within each sample's annotation [10]. Random subsets of 10, 25, 50, 100, and 200 arrays were selected from the available 491 arrays and used to train refRMA models. Each was applied to an exclusive test set of 50 arrays from the same normal GEO population. Note that, as the training set size increases, the more likely the 50 test arrays will have common characteristics. Classic RMA probe set summaries were also created using these same 50 arrays. Correlation of each test sample summarized by refRMA was calculated relative to the same test sample summarized by Classic RMA (i.e., trained on the 50 test set samples) using all probe sets. The mean correlation across the 50 test samples was then calculated. This process was repeated 100 times using random selection of both training and test sets to yield correlation distributions. Boxplots of the correlation distributions relative to training set size are shown in Figure 2. The Full refRMA model (noted in the plot as "DB") was also compared to Classic RMA using the same 50 randomly selected test sets over 100 iterations.

As expected, the correlation to Classic RMA increases as the refRMA training set size increases until correlations approaching 1 are achieved. The Full refRMA model also approaches 100% correlation. The plot also indicates differences in correlation as a function of independence of training and test set arrays. As the GEO-based refRMA models randomly select from the same population as that of the 50 test set arrays, there are likely to be many common biological and technical factors such as microarray processing site, replicate arrays from the same treatment or disease group, etc. The Full refRMA represents a more independent training set. The slight drop in correlation suggests that this effect is observable, but not of substantial consequence.

The results generally indicate that the Full refRMA model can be applied to data external to Gene Logic's processes with good confidence.

**Figure 3:**
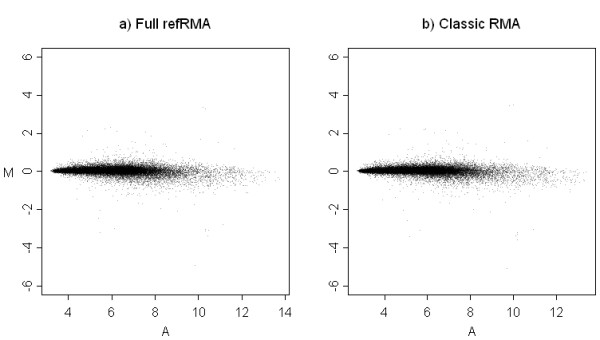
**MA plots for Classic RMA and refRMA models. **Mean probe set intensities as summarized for each of two sets of 15 normal liver samples are shown. The a) Full refRMA model was calculated based on 1,614 biologically diverse samples exclusive of the 30 normal liver test set samples contributing to this plot. The b) Classic RMA model was trained using only the 30 test set samples. Consistency in general data structure is observed regardless of training set. For each probe set, the mean intensity was calculated by averaging individual expression values across the 15 samples in each set.

Data structure was assessed after application of separate normalization schemes using 2 exclusive sets of 15 liver normal samples from Gene Logic's BioExpress^® ^database that were not used to build the Full refRMA model. For each probe set, the mean intensity was calculated by averaging individual expression values across the 15 samples in each set. MA plots of the mean probe set intensities are shown in Figure 3 for the Full refRMA and Classic models. The plots indicate the same general structure of data with similar distributions of both signal (M) and variability (A).

**Figure 4:**
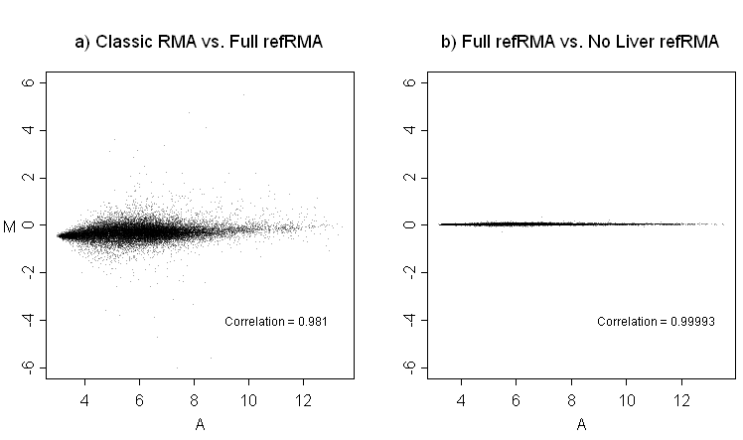
**MA plots showing differences between Classic and refRMA models for the same test set. **Mean probe set intensities as summarized by the two different RMA models are shown for the same test set of 15 normal liver samples. In each case, the y-axis (i.e., the axis indicating variability) is scaled relative to the biological variability observed in Figure 3 in order to contextualize the effect of model relative to effect of inherent variability contributed by different test set sampling. a) Classic and refRMA models differ somewhat, while b) the Full refRMA model and a refRMA model where liver has not been used in the training set are almost identical, indicating that the Full refRMA model is unaffected by incorporation of other single organs. For each probe set, the mean was calculated by averaging individual expression values across the 15 samples in each set.

MA plots of the mean probe set intensities were also constructed for comparisons of a single set of 15 normal liver samples summarized using either Classic RMA, Full refRMA, or a similar version of the Full refRMA where all liver samples have been replaced with samples from other organs. The results are shown in Figure 4 using y-axis scales based on the spread of data dictated by the biological variability from the Figure 3 MA plots. The comparison of Classic RMA to Full refRMA results in an overall correlation over mean probe set intensities of 0.981 and shows some spread of data primarily at the low end of expression. A slight systematic shift is observed at the low end of expression. It is not clear what this shift is due to, but is an indication of subtle systematic differences between small and large training set size models. The comparison of the Full refRMA and liver-naïve refRMA models results in an overall correlation of mean probe set intensities of 0.99993. This high degree of correlation is most likely due to the low percentage of data removed from the training set for the liver-naive refRMA model and suggests stability by design. Therefore, almost identical probe set intensities result from a model either built with or without any single organ.

**Figure 5:**
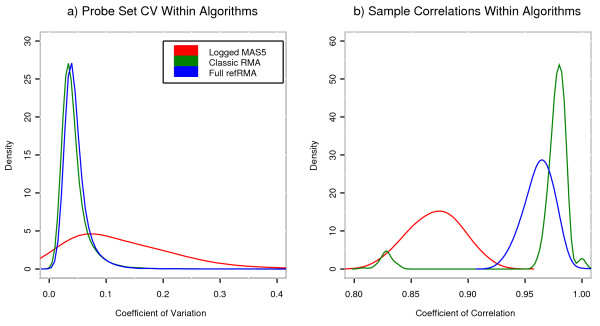
**Coefficient of Variation and Correlations for summarization methods. **The distributions of a) probe set-specific coefficients of variation resulting from log_2 _MAS5, Classic RMA, and Full refRMA summarization schemes on 30 liver normal samples and b) correlations of all possible pair-wise comparisons across samples using all probe sets on the HG U133A GeneChip. The two small bumps in the correlation Classic RMA plot are discussed in the manuscript body.

Histograms for coefficients of correlations and variation are shown in Figure 5 according to the Classic RMA and Full refRMA workflows. The correlation histogram is based on all possible pair-wise sample comparisons across 30 normal liver samples that were not used to build the Full refRMA model. The covariance histogram is based on each probe set's covariance across these same 30 normal liver samples.

The histograms indicate almost identical distributions for both probe set-based variation and sample-based correlation for both the Classic RMA and Full refRMA workflows with a slight unfavorable shift for Full refRMA compared to Classic RMA. This small shift can be viewed as the necessary "cost" associated with the use of a static normalization scheme for true biological samples. Two small "bumps" in the distribution of Classic RMA correlations are evident. The bump centered at 0.83 is explained by a single sample's correlation to the rest of the data set. We were not able to explain the smaller bump centered close to 1. Interestingly, both are resolved by the refRMA model, which indicates potential differences between the two models. Unfortunately, it is not clear as to whether this is a case of Full refRMA more successfully dealing with technical artifacts on the chip or if it is a case of Full refRMA diluting true biological effects.

Fold change and p-value conservation is important to establish with any summarization algorithm. Although it cannot be expected that different normalization and summarization techniques will select exactly the same probe sets, our goal is to show that a large number of probe sets are selected with both the Classic RMA and Full refRMA normalization schemes.

Consistency of regulation events was compared for the case where the same model was used to summarize different test sets of 15 normal vs. 15 malignant liver samples using 20 bootstrapping iterations with sample replacement and for the case where Classic RMA and Full refRMA were used to summarize the same test set of 15 normal vs. 15 malignant liver samples [11].

**Figure 6:**
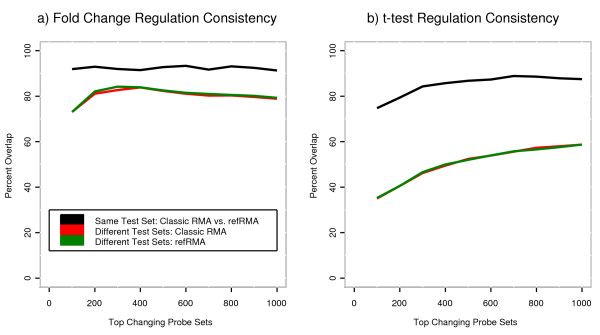
**CAT plots showing the overlap of top *n *selected probe sets. **Two types of comparisons are made for each of the a) fold change and b) t-test metrics frequently used for selection of regulated probe sets within a simple experimental design such as the one used here (i.e., 15 normal vs. 15 malignant liver samples). For both fold change and t-test metrics, the consistency of regulation events is based on overlapping selection of top *n *regulated probe sets. First, multiple test sets of 15 normal and 15 malignant samples are used to assess consistency of regulation using the same summarization algorithm over 20 bootstrap iterations with sample replacement. Second, the same test set of 15 normal and 15 malignant samples are used to assess consistency of regulation using different summarization schemes, namely Classic vs. Full refRMA. For each metric, regulation consistency due to summarization scheme is higher than consistency due to different test sets

Results are shown in Figure 6 as a function of increasing thresholds of top regulated probe sets, also known as a "correspondence at the top" (CAT) plot more fully described in Irizarry, et al [12]. For both fold change and t-test metrics, the consistency of regulation events based on selection of top regulated probe sets is higher for Classic RMA vs. Full refRMA for the same test set than when different test sets are summarized by the same model. This indicates that the variability associated with different test sets of arrays is higher than that of different summarization models and constitutes a positive outcome for the Full refRMA model as a viable summarization technique. In addition, Full refRMA and Classic RMA yield approximately the same degree of regulation consistency for both metrics when challenged with different test sets.

## Discussion

One of the limitations with the Classic RMA workflow and other types of multiple array-dependent normalization and summarization schemes is that the resulting models are usually applied to the same samples that were used in its training. This tight dependency between the training and test sets results in a situation where the probe set data cannot be archived in a continuously updated database in the absence of a static normalization scheme.

To counter this limitation, we have used the extensive content from Gene Logic's BioExpress^® ^reference database of human samples to build a static normalization scheme that can be applied to incoming human data from the HG U133A array on a continual basis. We have shown that a Full refRMA model, trained on a widely varying number of biologically distinct samples, results in similar probe set intensities compared to the classic RMA workflow as far as general data structure, similarity metrics, and number of regulation events. We have also shown that the Full refRMA model is applicable to data external to the processes underlying the BioExpress^® ^database.

Our conclusion from the above observations is that the Full refRMA model can generally be used to summarize an archival database on an ongoing basis for incoming samples. By applying the model to all samples, we can create a probe set-level expression database that is both consistent across organ and pathology category and provides the inherent benefits of RMA summarization. This same observation may be transitive across other multi-array summarization algorithms such as gcRMA, MBEI, and PLIER or variants thereof. The appropriate reference objects would need to be trained and provided for each of these according to their basic input requirements.

Reference database normalization schemes such as the Full refRMA model described can also be applied to samples in an *ad hoc *manner (i.e., for purposes other than establishing a reference database). Analysts can use the normalization schemes on single sample set activities. A number of logistical advantages are inherent within such a workflow.

First, a pre-computed reference model can be used to alleviate memory-intensive calculations for studies of large sample sizes. This issue has been noted previously and has been addressed by re-sampling and partitioning methods [13]. Models such as RMA increase memory usage relative to the number of input samples for its training such that desktop computing may not be possible for sample sizes in the hundreds or thousands. Pre-calculated models of appropriate training set scope would allow for a close approximation of the sample set-specific model, while circumventing memory constraints.

Second, any one of a number of the models could eventually be used as a standardization mechanism across the industry if a large number of users find such models applicable and valid for their data sets. Obviously, the degree of validation necessary for such an "industry-universal" model is extensive and is outside the scope of this publication. However, given the recent emphasis on microarray analysis standards, the development of a static normalization scheme on a widely varying training set for such algorithms as RMA is a useful starting tool.

Despite attempts to maximize the applicability of any reference model for the multi-array algorithms, there are potential technical and biological variables which may degrade their performance. Microarray data generation protocols are comprised of many hands-on technical processes and reagents. The combination of all possible variants of each step and types of reagents prohibits claiming that any single model is robust for all variables. Sources of technical variability include different labeling technologies, PMT settings, human operators, RNA quality, reagent lots/manufacturer, and innate day-to-day variables. Despite this multi-step process, technical variability has been reported to be among the smallest variability factors within organ systems in human and mouse, although these studies have not attempted to quantify individual technical factors and have not assessed cross-laboratory factors [14,15]. In our own experience, biological variability according to organ and pathological category is rather large, while technical variation is relatively small. The results of the GEO-based assessment suggest that appropriate summarization across array processing centers is supported by the Full refRMA model.

In addition to normal technical variability associated with microarray data generation, there are more extreme deviations in sample preparation associated with small- or micro-sample amplification, laser-capture microdissection techniques, and multi-gene knockout experiments that may perturb the probe-level signal distributions beyond what is reasonable for a conventional training set. The ability of the Full refRMA model to properly summarize these types of purposeful deviations has not been investigated and cannot be claimed. This concern is also generally applicable to the other multiple array-dependent algorithms.

Biological variables such as organ, pathology state, gender, age, race, and others are well represented within the training set of 1,614 unique samples and the Full refRMA model should be relatively robust for these factors. We have demonstrated that samples from an organ naïve to a large-sample refRMA training set are normalized almost identically to a large-sample refRMA training set where the organ is well-represented.

Finally, it should be noted that most applications of the RMA workflow involve a relatively small number of experiments (n < 50) obtained directly from .cel file data. We believe that the Classic RMA workflow should be utilized for these situations whenever possible due to the optimal applicability of a model to its component samples. The refRMA workflow is valuable for situations where summarized expression data is archived within large, continually updated enterprise database systems or for alleviating memory constraints for experiments of large size, but in no way substitutes for the Classic RMA workflow when study designs permit the use of the latter.

## Conclusion

In conclusion, a primary logistical issue associated with multiple array-dependent normalization and summarization algorithms is their reliance on a limited set of arrays to produce a model that is specific to that set. A reference model based on a training set comprised of a large, biologically diverse training set has been developed for the HG U133A GeneChip^® ^arrays such that it can be applied to a wide range of sample organ types and pathology states.

## Methods

### refRMA algorithm and workflow

The refRMA algorithm is a version of the log scale linear additive RMA procedure as described in Irizarry, et al. [1] and is explained as follows within that manuscript: "The model can be written as *T*(*PM*_*ij*_) = *e*_*i *_+ *a*_*j *_+ *ε*_*ij*_, *i *= 1,..., *I*, *j *= 1,..., *J*, where *T *represents the transformation that background corrects, normalizes, and logs the PM intensities, *e*_*i *_represents the log_2 _scale expression value found on arrays *i *= 1,..., *I*, *a*_*j *_represents the log scale affinity effects for probes *j *= 1,..., *J*, and *ε*_*ij *_represents error..." The original implementation of RMA uses multiple array information in two ways. The first is through quantile normalization, which uses all available arrays to form an average empirical distribution. Normalization is achieved by forcing all arrays to have this distribution [6]. The second comes from fitting the linear model for each probe set across all available arrays. The original implementation uses median polish. In this article we will refer to this workflow as "Classic RMA". Classic RMA can be thought of as a one-step process given that any implemented script or code base can assume a single sample set input and an immediate output in the form of a single matrix of probe set expression values for each sample set. Persistence of intermediate data objects or other outputs is not necessary using the Classic RMA scheme.

The refRMA workflow utilizes the above model, but uses a predetermined group of arrays to estimate the probe effects and the average empirical distribution to be used with quantile normalization. Specifically, the workflow can be described as two separate processes, a training process and an application process. The training process accepts a single data set (i.e., set of arrays) as input and outputs two data objects which will be persistently archived: 1) a "probe effect" vector is compiled based on the individual log scale probe affinity effects, *a*_*j*_, with vector length equivalent to the number of probes on the array, and 2) a "normalization" vector, also of length equivalent to the number of probes on the array, is compiled based on the intensity, *T(PM_ij_)*, Note that this training portion of the refRMA workflow is exactly as specified by the Classic RMA model and existing BioConductor code within the affy package can be utilized to support it [17].

The inputs for the application portion of the workflow are the two archived vectors and a single sample run on the same array type. The normalization and probe effect vectors are applied to the test sample, as defined by the model, to calculate probe level intensity values. Note that the application of these two static vectors to the test sample constitutes a one-way function in which the test sample is normalized and summarized by both vectors, but does not modify either one. BioConductor code for the Classic RMA implementation can also be utilized for this portion of the workflow with a modification to utilize the archived vector objects from the training portion instead of the default intermediate objects. Note that there is no longer a group of samples to perform the full median polish summarization. Therefore, a median is taken across the resident probes of each probe set to establish probe set level summaries. The output is a vector of probe set expression values for the single sample. The application process can be repeated over each sample in a multiple array test set such that a single matrix of probe set expression values is derived. An important advantage of this procedure is that it does not require multiple arrays.

Note that, although this alternate workflow is defined for the RMA algorithm, a similar workflow can be defined for other multiple array-dependent algorithms.

## Availability

The HGU133A Full refRMA model will be provided for public use as a part of the Bioconductor project [16]. In the interim, the Full refRMA model is available by request from the corresponding author.

## Authors' contributions

SK trained the Full refRMA model and conducted all computational and statistical comparisons involving liver samples. RAI performed the computational and statistical comparison of refRMA models to publicly available data sets. XL performed the affycomp assessment. MT coded the refRMA workflow into R and prepared the code for use by the general public. MWP compiled and drafted the full manuscript and developed the training and testing strategies supporting the manuscript. All authors read and approved the final manuscript.

## Supplementary Material

Additional File 1 The numbers of samples used to train the Full refRMA model based on organ and pathology category. Click here for file
